# Hybrid TSR–PSR Alternate Energy Harvesting Relay Network over Rician Fading Channels: Outage Probability and SER Analysis

**DOI:** 10.3390/s18113839

**Published:** 2018-11-09

**Authors:** Tan N. Nguyen, Phu Tran Tin, Duy Hung Ha, Miroslav Voznak, Phuong T. Tran, Minh Tran, Thanh-Long Nguyen

**Affiliations:** 1 VSB-Technical University of Ostrava 17. listopadu 15/2172, 708 33 Ostrava-Poruba, Czech Republic; nguyennhattan@tdtu.edu.vn (T.N.N.); phutrantin@iuh.edu.vn (P.T.T.); haduyhung@tdtu.edu.vn (D.H.H.); miroslav.voznak@vsb.cz (M.V.); 2 Wireless Communications Research Group, Faculty of Electrical and Electronics Engineering, Ton Duc Thang University, Ho Chi Minh City, Vietnam; 3 Optoelectronics Research Group, Faculty of Electrical and Electronics Engineering, Ton Duc Thang University, Ho Chi Minh City, Vietnam; tranhoangquangminh@tdtu.edu.vn; 4 Center for Information Technology, Ho Chi Minh City University of Food Industry, Ho Chi Minh City, Vietnam; longthng@gmail.com

**Keywords:** half-duplex, amplify-and-forward (AF), outage probability, TSR–PSR, SER

## Abstract

In this research, we investigate a hybrid time-switching relay (TSR)–power-splitting relay (PSR) alternate energy harvesting (EH) relaying network over the Rician fading channels. For this purpose, the amplify-and-forward (AF) mode is considered for the alternative hybrid time TSR–PSR. The system model consists of one source, one destination and two alternative relays for signal transmission from the source to the destination. In the first step, the exact and asymptotic expressions of the outage probability and the symbol errors ratio (SER) are derived. Then, the influence of all system parameters on the system performance is investigated, and the Monte Carlo simulation verifies all results. Finally, the system performances of TSR–PSR, TSR, and PSR cases are compared in connection with all system parameters.

## 1. Introduction

Currently, energy harvesting (EH) from green environmental sources and the conversion of this energy into the electrical energy used to supply communication network devices is considered to be a leading research direction. Furthermore, this solution can provide not only environmentally friendly energy supplies, but also self-maintained, long-lived, and autonomous communication systems. In the series of primary environmentally green energy sources, such as solar, wind, geothermal, and mechanical energy, radio frequency (RF) signals can be considered as a prospective energy source in the future. The RF sources can be used independently from time and location in urban areas and can be produced cheaply in small dimensions, which could be a significant advantage in the manufacturing of small and low-cost communication devices such as sensor nodes. Moreover, RF signals could provisionally fill the role of information transmission or energy harvesting in the sensor nodes. Wireless power transfer and the harvesting of electrical energy from a power source to one or more electrical loads is a well-known technique in communication networks. Thus, this research direction for RF-powered mobile networks has received significant attention during the last decade in wireless sensor networks (WSNs) and cooperative communication systems from both academia and industry [1,2,3,4,5,6,7,8,9]. Furthermore, EH wireless communication can substantially prolong the network lifetime for wireless sensor networks with low power nodes, and RF signals can recharge nodes more controllably. Wireless nodes can harvest RF energy either in the time domain before data reception, or in the power domain by dividing the received RF signals for the EH and information decoding (ID) [9,10,11,12]. In a cooperative network, references [13,14,15,16] developed two new relaying protocols based on the receiver structures adopted at R, termed time-switching-based relaying (TSR) and power-splitting-based relaying (PSR). From [14,15,16], the TSR and PSR protocols have some drawbacks; for instance, TSR loses information in the switching process to the harvesting mode, and PSR has a low coverage area. Furthermore, PSR requires a complicated hardware structure to make sure that a proper portion of energy from the source signal is extracted for energy harvesting. In contrast, TSR can simplify the hardware at the expense of the throughput or achievable rate of the system. Based on the fact that both TSR and PSR protocols have their drawbacks, the proposed method combines these two protocols to get the best out of them. This solution is obtained in this paper by using an adaptive relaying protocol [17,18]. From the other point of view, the authors highlighted the problem of maximizing the throughput in connection with power allocation and the degree of channel state information (CSI) in [19], and secrecy systems operating over spatially correlated composite fading channels are presented in [20]. Also, the performance of dynamic relays in different types of cellular networks under the presence of inter-cell interference (ICI) is investigated in [21], and a novel approach for maintaining the gains of relaying and keeping the signaling overheads at a low level is proposed in [22]. Furthermore, maximizing harvested power in MIMO SWIPT systems with PSR is investigated in [23], the joint design of spatial channel assignment and power allocation in MIMO systems is studied in [24], and ref. [25] considers a dual-hop cognitive inter-vehicular relay-assisted communication system by the double Rayleigh fading distribution.

The main objective of this paper is to provide a system performance analysis (in terms of the outage probability and symbol error ratio (SER)) of hybrid TSR–PSR alternate energy harvesting relaying networks over Rician fading channels. In the analysis process, we analyze and derive the exact and asymptotic expressions of the outage probability and SER. After that, the Monte Carlo simulation is used to validate the analytical analysis in connection with all system parameters. The main contributions of this research are as follows:

(1) We propose and investigate the alternative hybrid TSR–PSR energy harvesting relaying networks over Rician fading channels. In this model, two relays R1 and R2 are alternatively used for energy harvesting and data transmission process from S to D;

(2) The exact and asymptotic expressions of the outage probability and SER are proposed, analyzed and derived. Moreover, the comparison of the exact and asymptotic expressions of the outage probability and SER in three cases—TSR, PSR, and TSR–PSR—is demonstrated;

(3) The influence of all system parameters on the outage probability and SER is investigated and discussed;

(4) The Monte Carlo simulation is used to verify all the research results.

The rest of this manuscript is organized as follows. In Section 2, we present the proposed system model. Section 3 investigates the exact and asymptotic analysis of the outage probability and SER. Numerical results and a discussion are given in Section 4. Section 5 concludes this manuscript.

## 2. System Model

**Figure 1:**
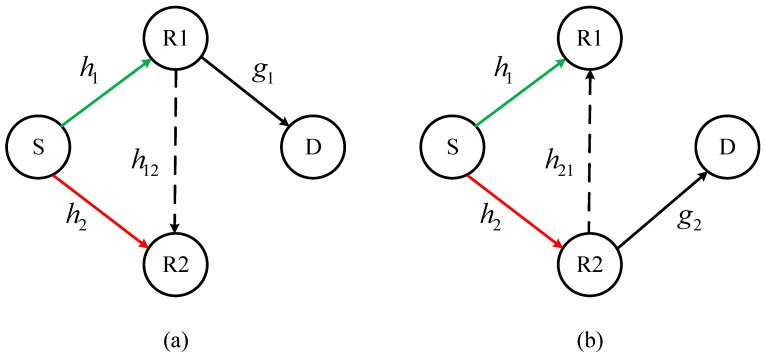
The system model. The green, red and black lines represent the first-hop and second-hop transmission, respectively. The green and dashed back lines represent the data transmission (DT) and energy harvesting (EH), respectively. (**a**) R_1_-DT and R_2_-EH; (**b**) R_2_-DT and R_1_-EH.

Consider an AF relaying system with four nodes: the source S, two relays R_1_, R_2_ and the destination D as shown in Figure 1. Each device works with a single antenna and in a half-duplex (HD) mode, and there is no direct link between R and D. In this model, S, and D have their own stable power supplies, while R1 and R2 operate with EH and alternately forward source data according to the AF protocol. We denote that *h*_1_ and *h*_2_ are the fading channel gains from the source to relays, *g*_1_ and *g*_2_ are the fading channel gain from the relays R_1_ and R_2_ to the destination D, and *h*_12_ and *h*_21_ are the gain factors between R_1_ and R_2_, respectively [26,27,28,29].

**Figure 2:**
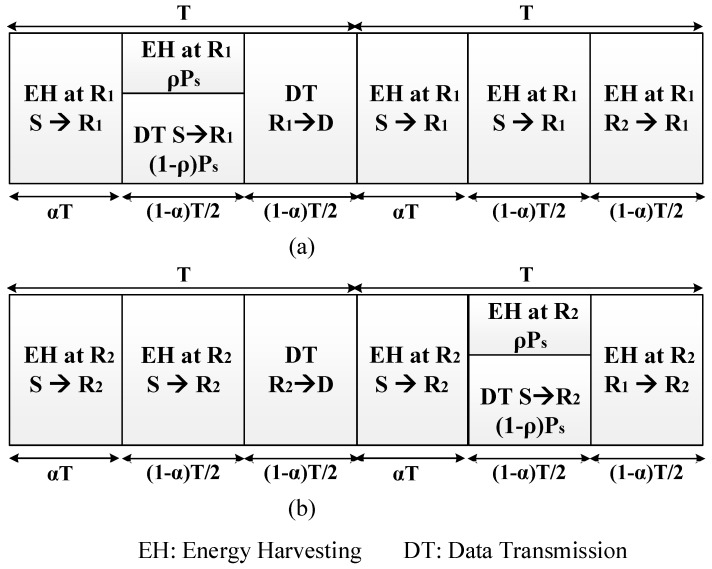
The information transmission and energy harvesting process. (**a**) R_1_-DT and R_2_-EH; (**b**) R_2_-DT and R_1_-EH.

Moreover, Figure 2 shows the division of the transmission time. In the first interval time αT with 0≤α<1, S transfers energy by signal to R_1_ and R_2_. After that, S transfers the part of energy $\rho P_{s}$ (0≤ρ<1) to R_1_ in the next (1−α)T/2 interval time and uses $(1-\rho )P_{s}$ energy to transfer information to R_1_. In the same interval time, R_2_ harvests energy from S. In the final (1−α)T/2 interval time, R_1_ transfers information to D and R_2_ harvests energy from R_1_ [14,25,26,29].

In this model, R_1_ forwards the source information data to D by using its energy harvested in the current T blocks and the previous T blocks. Please note that R_1_ and R_2_ always harvest energy from the received RF signals in all of the first T blocks. In the following T blocks, R_2_ works as a helping relay, while R_1_ harvests energy in all T blocks by overhearing the transmissions from S and R_2_. The EH and data relay of R_2_ are performed similarly to the above procedure for R_1_. Thus, R_1_ and R_2_ will alternately forward source data in every T block. Compared with the TS-EH or PS-EH-based single-relay system, more energy can be harvested by relays in our protocol for the DT [14,26,29].

## 3. System Performance

In this section, we analyze and investigate the energy harvesting and data transmission processes in the two relays in the hybrid TSR–PSR protocol. To increase the understanding of the readers, we show all symbols used in Table 1.

In the system model, the inter-relay channel is assumed to be symmetric, i.e., $h_{12}=h_{21}$. Rician block fading is assumed, so all the channels are circularly-symmetric jointly-Gaussian complex random and denoted as $h_{i}\approx CΝ\left(0,1\right)$, $g_{i}\approx CΝ\left(0,1\right)$ and $h_{12}\approx CΝ\left(0,1\right)$, where i∈(1,2).

In the hybrid TSR–PSR alternative relaying, the source provides an energy signal to both *R*_1_ and *R*_2_ in αT and (1−α)T/2 blocks. In the (1−α)T/2 block, R_1_ allocates 0≤ρ<1 (ρ is the power splitting factor) as part of the received source signal for the energy harvesting (EH). Therefore, the total harvested energy at R_1_ can be given by

(1) $$E_{r1a}=\eta \alpha TP_{s}+\frac{\eta \rho (1-\alpha )TP_{s}}{2}$$

where ($E\left\{{\left|h_{i}\right|}^{2}\right\}=1$ and $h_{i}\approx CΝ\left(0,1\right)$, $g_{i}\approx CΝ\left(0,1\right)$, $h_{12}\approx CΝ\left(0,1\right)$), 0<η<1 and 0≤α<1 are the energy conversion efficiency and time-switching factor, respectively.

In this model, the average EH amount by omitting the small-scale channel fading is proposed and considered.

In a similar way, the total harvested energy at R_2_ can be given by the equation below:

(2) $$E_{r2a}=\eta \alpha TP_{s}+\frac{\eta \rho (1-\alpha )TP_{s}}{2}$$

When S provides the data to R_1_, after splitting the ρ part of the received signal for the EH at the relays, the remaining signal at R_1_ can be obtained as

(3) $$y_{r1}=\sqrt{1-\rho }h_{1}x_{s}+n_{r1}$$

where $n_{r1}$ is the additive white Gaussian noise (AWGN) with variance *N*_0_ at R_1_, and $E\left\{{\left|x_{s}\right|}^{2}\right\}=P_{s}$ in which E{•} is expectation operator.

Furthermore, R_1_ amplifies and forwards the signal to D in the next stage. The transmitted signal from R_1_ can be expressed as

(4) $$x_{r1}=\beta y_{r1}$$

where $\beta =\frac{\sqrt{P_{r1}}}{\sqrt{(1-\rho )P_{s}{\left|h_{1}\right|}^{2}+N_{0}}}$ is the amplifying factor.

Then, the received signal at D can be formulated as the following expression:

(5) $$y_{1d}=g_{1}x_{r1}+n_{1d}$$

where $n_{1d}$ is the additive white Gaussian noise (AWGN) with variance *N*_0_ at D, $E\left\{{\left|x_{r1}\right|}^{2}\right\}=P_{r1}$, and $P_{r1}$ is the average transmitted power of R_1_.

Replacing (3) and (4) into (5), the received signal at D can be obtained as:

(6) $$y_{1d}=\beta g_{1}\left[\sqrt{1-\rho }h_{1}x_{s}+n_{r1}\right]+n_{1d}=\underset{signal}{\underset{︸}{\beta g_{1}\sqrt{1-\rho }h_{1}x_{s}}}+\underset{noise}{\underset{︸}{\beta g_{1}n_{r1}+n_{1d}}}$$

In this case, when R_1_ performs the delay-tolerant (DT) transmission mode, the end to end signal to noise ratio (SNR) at D can be calculated as

(7) $$\gamma_{e2e1}=\frac{E\left\{{\left|signal\right|}^{2}\right\}}{E\left\{{\left|noise\right|}^{2}\right\}}=\frac{\beta^{2}{\left|g_{1}\right|}^{2}{\left|h_{1}\right|}^{2}P_{s}(1-\rho )}{\beta^{2}{\left|g_{1}\right|}^{2}N_{0}+N_{0}}$$

After algebra calculation and using the fact that *N*_0_ << *P_r_*, the end to end SNR can be obtained:

(8) $$\gamma_{e2e1}=\frac{(1-\rho )P_{s}P_{r1}{\left|h_{1}\right|}^{2}{\left|g_{1}\right|}^{2}}{{\left|g_{1}\right|}^{2}N_{0}+(1-\rho )P_{s}{\left|h_{1}\right|}^{2}N_{0}}$$

In this T block time, R_2_ can harvest energy from S in αT+(1−α)T/2 blocks, i.e., $\frac{T}{2}(1+\alpha )$ blocks, and R_2_ can also harvest energy from R_1_ in (1−α)T/2 blocks. Therefore, the total harvested energy at R_2_ when R_1_ joins in the data transmission (DT) can be calculated by

(9) $$E_{r2b}=\frac{\eta (1+\alpha )TP_{s}}{2}+\frac{\eta (1-\alpha )TP_{r1}}{2}$$

Similar to R_2_, the total harvested energy at R_1_ when R_2_ joins in the DT can be obtained as

(10) $$E_{r1b}=\frac{\eta (1+\alpha )TP_{s}}{2}+\frac{\eta (1-\alpha )TP_{r2}}{2}$$

where $P_{r2}$ is the average transmitted power of R_2_.

From the EH process at R_1_ in the previous T blocks and current T blocks, the total harvested energy of R_1_ for DT can be obtained as

(11) $$\begin{array}{cc} E_{r1} & =E_{r1a}+E_{r1b}=\eta \alpha TP_{s}+\frac{\eta \rho (1-\alpha )TP_{s}}{2}+\frac{\eta (1+\alpha )TP_{s}}{2}+\frac{\eta (1-\alpha )TP_{r2}}{2}\\ & =\frac{\eta T\left[(3\alpha -\alpha \rho +1)P_{s}+\rho +(1-\alpha )P_{r2}\right]}{2} \end{array}$$

because of the symmetry property in our proposed system. Similar to R_1_, the total harvested energy of R_2_ also can be obtained as the following:

(12) $$E_{r2}=E_{r2a}+E_{r2b}=\frac{\eta T\left[(3\alpha -\alpha \rho +1)P_{s}+\rho +(1-\alpha )P_{r1}\right]}{2}$$

From (11), the average transmitted power of R_1_ can be calculated as

(13) $$P_{r1}=\frac{E_{r1}}{(1-\alpha )T/2}=\frac{\eta T\left[(3\alpha -\alpha \rho +1)P_{s}+\rho +(1-\alpha )P_{r2}\right]}{(1-\alpha )T}=\eta \left[\frac{(3\alpha -\alpha \rho +1)P_{s}+\rho }{1-\alpha }+P_{r2}\right]$$

From (12), the average transmitted power of R_2_ also can be obtained as

(14) $$P_{r2}=\frac{E_{r2}}{(1-\alpha )T/2}=\eta \left[\frac{(3\alpha -\alpha \rho +1)P_{s}+\rho }{1-\alpha }+P_{r1}\right]$$

Substituting (14) into (13), we obtain

(15) $$P_{r1}=\frac{\eta \Psi }{1-\eta }$$

where we denote $\Psi =\frac{(3\alpha -\alpha \rho +1)P_{s}+\rho }{1-\alpha }$.

Finally, the SNR of the proposed system in (7) can be rewritten as the following:

(16) $$\gamma_{e2e1}=\frac{(1-\rho )P_{s}P_{r1}{\left|h_{1}\right|}^{2}{\left|g_{1}\right|}^{2}}{{\left|g_{1}\right|}^{2}N_{0}+(1-\rho )P_{s}{\left|h_{1}\right|}^{2}N_{0}}=\frac{(1-\rho )P_{s}P_{r1}\varphi_{1}\varphi_{2}}{\varphi_{2}N_{0}+(1-\rho )P_{s}\varphi_{1}N_{0}}$$

where $\varphi_{1}={\left|h_{1}\right|}^{2},\;\varphi_{2}={\left|g_{1}\right|}^{2}$ and $P_{r1}$ is defined by (15).

### 3.1. Exact Outage Probability Analysis

The probability density function (PDF) of random variable (RV) $\varphi_{i}$ (where *i* = 1, 2) as in [28] is

(17) $$f_{\varphi_{i}}(x)=a\sum_{l=0}^{\infty }\frac{{\left(bK\right)}^{l}}{{\left(l!\right)}^{2}}x^{l}e^{-bx}$$

where we denote $\varphi_{1}={\left|h_{1}\right|}^{2},\;\varphi_{2}={\left|g_{1}\right|}^{2}$, $a=\frac{(K+1)e^{-K}}{\lambda_{i}},\;b=\frac{K+1}{\lambda_{i}}$, in which $\lambda_{i}$ is the unit mean value of RV $\varphi_{i}$ where *i* = 1, 2, respectively, because we consider the small-scale power fading ${\left|h_{1}\right|}^{2},\;{\left|g_{1}\right|}^{2}$ in the derivation. Therefore, *a* and *b* can be re-denoted as $a=(K+1)e^{-K},b=K+1$, *K* is the Rician K-factor defined as the ratio of the power of the line-of-sight (LOS) component to the scattered components.

The cumulative density function (CDF) of RV $\varphi_{i}$, where *i* = 1, 2, can be computed as in [17]:

(18) $$F_{\varphi_{i}}(\varsigma )=\int_{0}^{\varsigma }f_{\varphi_{i}}(x)dx=1-\frac{a}{b}\sum_{l=0}^{\infty }\sum_{n=0}^{l}\frac{K^{l}b^{n}}{l!n!}\varsigma^{n}e^{-b\varsigma }=1-\sum_{l=0}^{\infty }\sum_{n=0}^{l}\frac{K^{l}b^{n}e^{-K}}{l!n!}\varsigma^{n}e^{-b\varsigma }$$

(19) $$F_{\varphi_{i}}(\varsigma )=\int_{0}^{\varsigma }f_{\varphi_{i}}(x)dx=1-\frac{a}{b}\sum_{l=0}^{\infty }\sum_{n=0}^{l}\frac{K^{l}b^{n}}{l!n!}\varsigma^{n}e^{-b\varsigma }=1-\sum_{l=0}^{\infty }\sum_{n=0}^{l}\frac{K^{l}b^{n}e^{-K}}{l!n!}\varsigma^{n}e^{-b\varsigma }$$

**Theorem 1** (Exact Outage Probability)**.** *The expression of the exact outage probability of the proposed system can be formulated by the following:*

(20) $$\begin{array}{l} P_{out}^{1}=1-2ae^{-K}e^{-\frac{b\gamma_{th}N_{0}}{P_{r1}P_{s}(1-\rho )}}e^{-\frac{b\gamma_{th}N_{0}}{P_{r1}}}\sum_{l=0}^{\infty }\sum_{m=0}^{\infty }\sum_{n=0}^{l}\sum_{k=0}^{n+m}\frac{K^{l+m}b^{n+m}{\left(\gamma_{th}N_{0}\right)}^{n+m+1}(n+m)!}{l!{\left(m!\right)}^{2}n!k!(n+m-k)!P_{r1}{}^{n+m+1}{\left[P_{s}(1-\rho )\right]}^{\frac{2n+m-k+1}{2}}}\\ \times K_{m-k+1}\left(\frac{2b\gamma_{th}N_{0}}{P_{r1}\sqrt{P_{s}(1-\rho )}}\right) \end{array}$$

*where $K_{v}(•)$ is the modified Bessel function of the second kind and vth order*.

**Proof** **of Theorem 1.** See Appendix A. □

### 3.2. Asymptotic Outage Probability Analysis

From (16), at the high SNR regime, the end to end SNR can be approximated as

(21) $$\gamma_{\gamma_{e2e1}}^{\infty }=\frac{(1-\rho )P_{s}P_{r1}\varphi_{1}\varphi_{2}}{\varphi_{2}N_{0}+(1-\rho )P_{s}\varphi_{1}N_{0}}\approx \frac{P_{r1}\varphi_{2}}{N_{0}}$$

Then, the asymptotic outage probability can be formulated as

(22) $$P_{out}^{1,\infty }=\mathrm{Pr}\left(\frac{P_{r1}\varphi_{2}}{N_{0}}<\gamma_{th}\right)=\mathrm{Pr}\left(\varphi_{2}<\frac{\gamma_{th}N_{0}}{P_{r1}}\right)=1-\sum_{l=0}^{\infty }\sum_{n=0}^{l}\frac{K^{l}b^{n}e^{-K}}{l!n!}{\left(\frac{\gamma_{th}N_{0}}{P_{r1}}\right)}^{n}e^{-\frac{b\gamma_{th}N_{0}}{P_{r1}}}$$

### 3.3. SER (Symbol Error Ratio) Analysis

In this section, we obtain new expressions for the symbol error ratio (SER) at the destination. We first consider the outage probability, which was obtained in [30,31]. Thus, we obtain

(23) $$SER_{1}=E\left[\phi Q\sqrt{2\theta \gamma_{e2e1}}\right]$$

$Q(t)=\frac{1}{\sqrt{2\pi }}\int_{t}^{\infty }e^{-x^{2}/2}dx$ is the Gaussian Q-function, ω and θ are constants which are specific for the modulation type. (ϕ,θ)=(1,1) for BPSK and (ϕ,θ)=(1,2) for QPSK. For this purpose, the distribution function of $\gamma_{e2e1}$ is considered for analyzing the SER performance. Then, Equation (22) can be rewritten directly regarding the outage probability at the source by using integration as follows:

(24) $$SER_{1}=\frac{\phi \sqrt{\theta }}{2\sqrt{\pi }}\int_{0}^{\infty }\frac{e^{-\theta x}}{\sqrt{x}}F_{\gamma_{e2e1}}(x)dx$$

**Theorem 2** (Exact SER)**.** *The exact SER can be calculated by the below expression:*

(25) $$\begin{array}{l} SER_{1}=\frac{\phi }{2}-ae^{-K}\phi \sqrt{\theta }\sum_{l=0}^{\infty }\sum_{m=0}^{\infty }\sum_{n=0}^{l}\sum_{k=0}^{n+m}\frac{4^{m-k+1}K^{l+m}b^{n+2m-k+1}(n+m)!{\left(N_{0}\right)}^{n+2m-k+2}}{l!{\left(m!\right)}^{2}n!k!(n+m-k)!P_{r1}{}^{n+2m-k+2}{\left[P_{s}(1-\rho )\right]}^{\frac{2n+3m-3k+3}{2}}}\\ \times \frac{1}{{\left[\theta +\frac{3bN_{0}}{P_{r1}\sqrt{P_{s}(1-\rho )}}+\frac{bN_{0}}{P_{r1}}\right]}^{n+2m-k+5/2}}\times \frac{\Gamma \left(n+2m-k+\frac{5}{2}\right)\Gamma \left(n+k+\frac{1}{2}\right)}{\Gamma \left(n+m+2\right)}\\ \times F\left(n+2m-k+\frac{5}{2},m-k+\frac{3}{2};n+m+2;\frac{\theta -\frac{bN_{0}}{P_{r1}\sqrt{P_{s}(1-\rho )}}+\frac{bN_{0}}{P_{r1}}}{\theta +\frac{3bN_{0}}{P_{r1}\sqrt{P_{s}(1-\rho )}}+\frac{bN_{0}}{P_{r1}}}\right) \end{array}$$

*where Γ(•) is the gamma function, and F(υ,β;γ;z) is a hypergeometric function*.

**Proof** **of Theorem 2.** See Appendix B. □

**Theorem 3** (Asymptotic SER Analysis)**.** *The asymptotic SER can be formulated by the below equation:*

(26) $$SER_{1}^{\infty }=\frac{\phi }{2}-\frac{e^{-K}\phi \sqrt{\theta }}{2\sqrt{\pi }}\sum_{l=0}^{\infty }\sum_{n=0}^{l}\frac{K^{l}b^{n}}{l!n!}{\left(\frac{N_{0}}{P_{r1}}\right)}^{n}{\left(\theta +\frac{bN_{0}}{P_{r1}}\right)}^{-n-1/2}\Gamma \left(n+\frac{1}{2}\right)$$

**Proof** **of Theorem 3.** See Appendix C. □

## 4. Numerical Results and Discussion

For validation of the correctness of the derived outage probability and SER expressions, as well as the investigation of the effect of various parameters on the system performance, a set of Monte Carlo simulations are conducted and presented in this section. For each simulation, we first provide the graphs of the outage probability and SER obtained by the analytical formulas. Secondly, we plot the same outage probability and SER curves that result from the Monte Carlo simulation. To do this, we generate 10^6^ random samples of each channel gain, which are Rician distributed. The analytical curve and the simulation curve should match to verify the correctness of our analysis. All simulation parameters are listed in Table 2.

**Figure 3:**
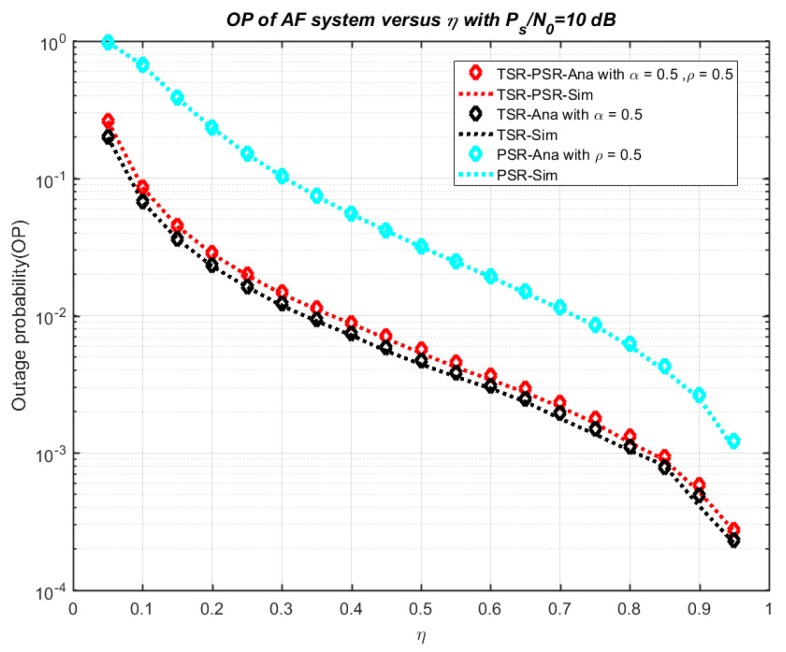
Outage probability versus η.

**Figure 4:**
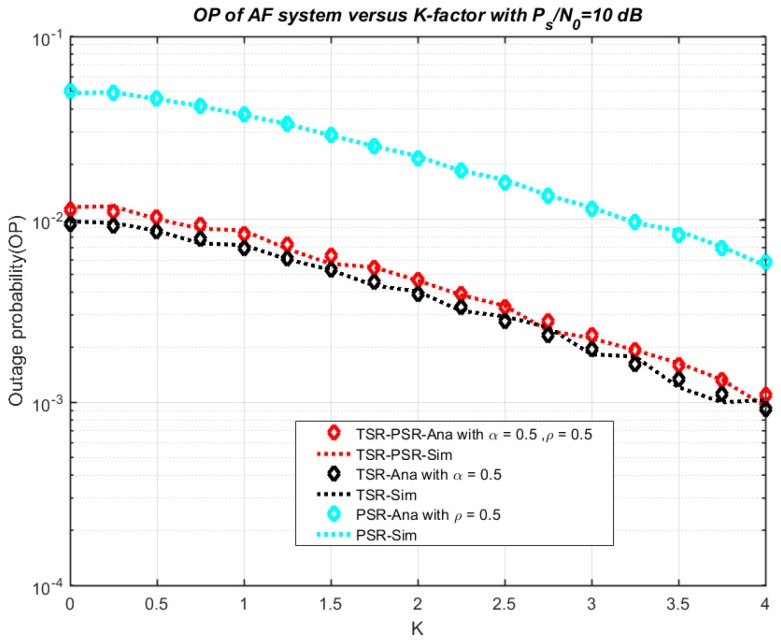
Outage probability versus K.

Figure 3 shows the outage probability of the model system versus η in three cases—TSR, PSR, TSR–PSR. In this model, we set *P_s_*/*N*_0_ = 10 dB, *ρ* = 0.5 and *α* = 0.5. From the results, we see that the outage probability decreases remarkably while η varies from 0 to 1. The research results show that the numerical results and simulation results match exactly with each other, validating the correctness of the theoretical analysis in the above section. Furthermore, the function of the outage probability to K is presented in Figure 4. Similarly, we set *P_s_*/*N*_0_ = 10 dB, *ρ* = 0.5 and *α* = 0.5, and the outage probability decreases remarkably while K varies from 0 to 4. Once again, the simulation results and theoretical results agree well with each other.

**Figure 5:**
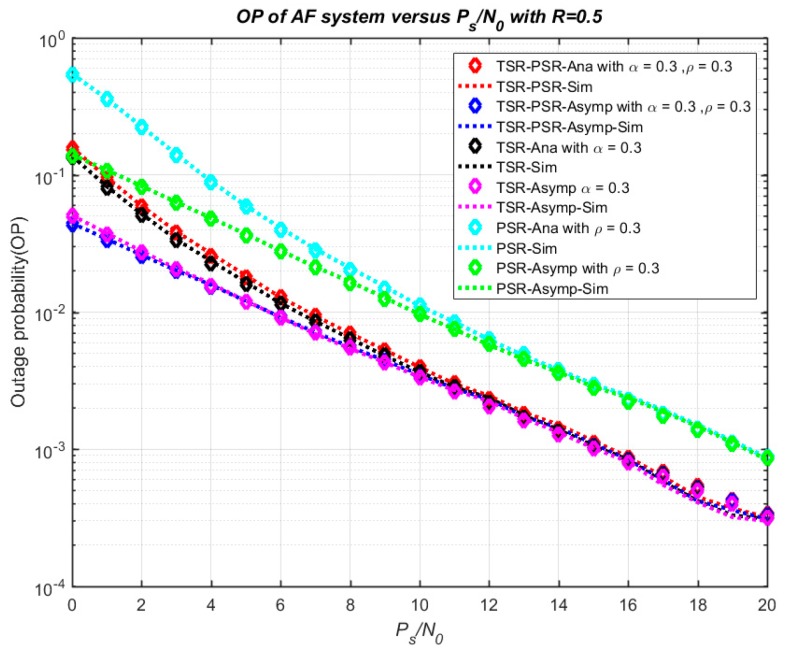
Outage probability versus *P_s_*/*N*_0_.

**Figure 6:**
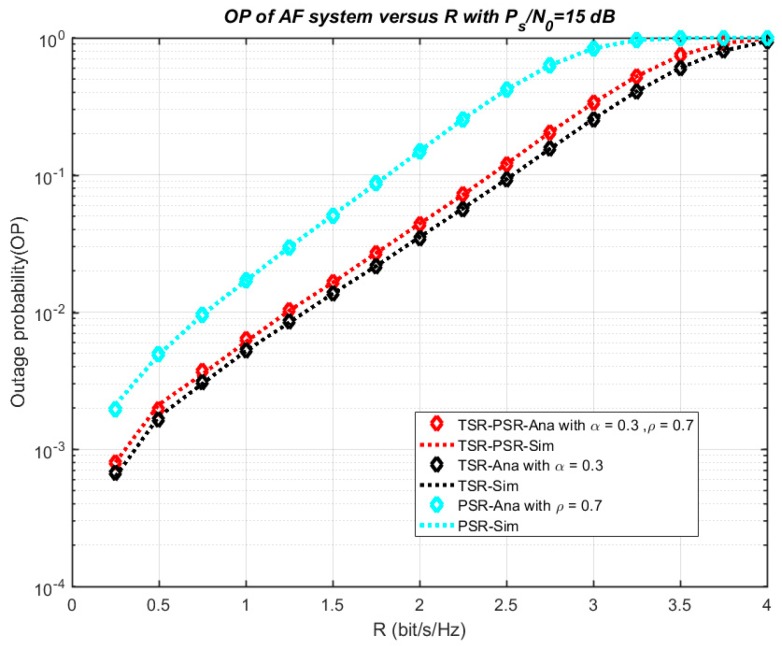
Outage probability versus R.

Figure 5 plots the numerical and simulation results of the system outage probability in connection with the ratio *P_s_*/*N*_0_. In Figure 5, both the exact and asymptotic outage probability in cases TSR, PSR, and TSR–PSR are illustrated. The main parameters are set as R = 0.5, *ρ* = 0.3 and *α* = 0.3. From the results, the exact outage probability decreases and comes close to the asymptotic line when the ratio *P_s_*/*N*_0_ increases from 0 to 20 dB. On another hand, the influence of R on the outage probability in three cases—TSR, PSR, TSR–PSR—is investigated in Figure 6 with *P_s_*/*N*_0_ = 15 dB, *ρ* = 0.7 and *α* = 0.3. The outage probability significantly increases with R from 0 to 4. From Figure 5 and Figure 6, the analytical results and the simulation results match well with each other for all values of R and *P_s_*/*N*_0_.

**Figure 7:**
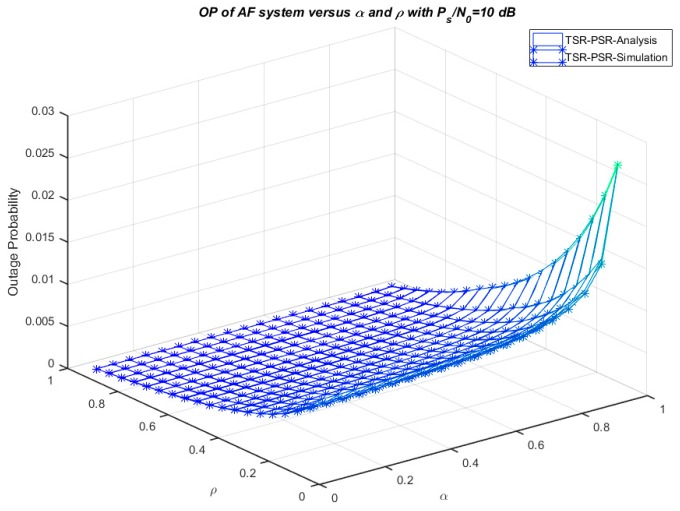
Outage probability versus *ρ* and *α*.

**Figure 8:**
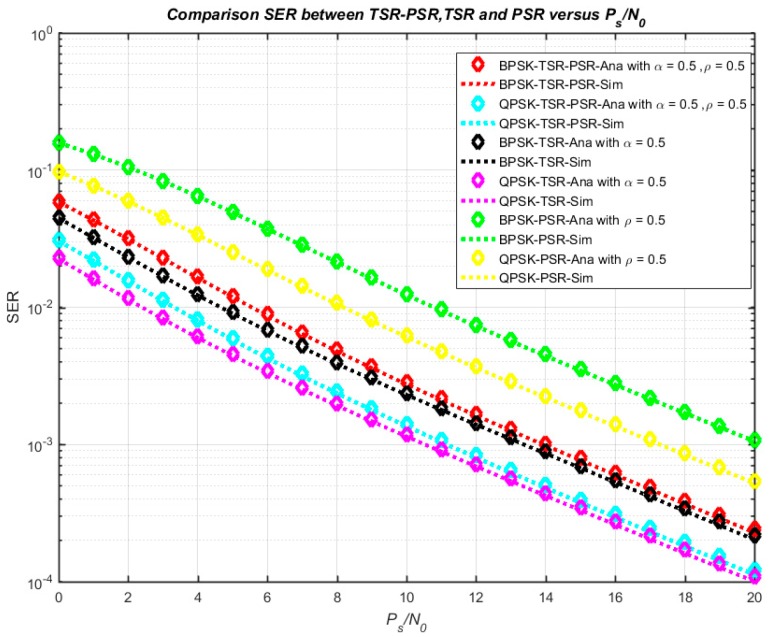
SER versus *P_s_*/*N*_0_ in the cases TSR, PSR, and TSR–PSR.

**Figure 9:**
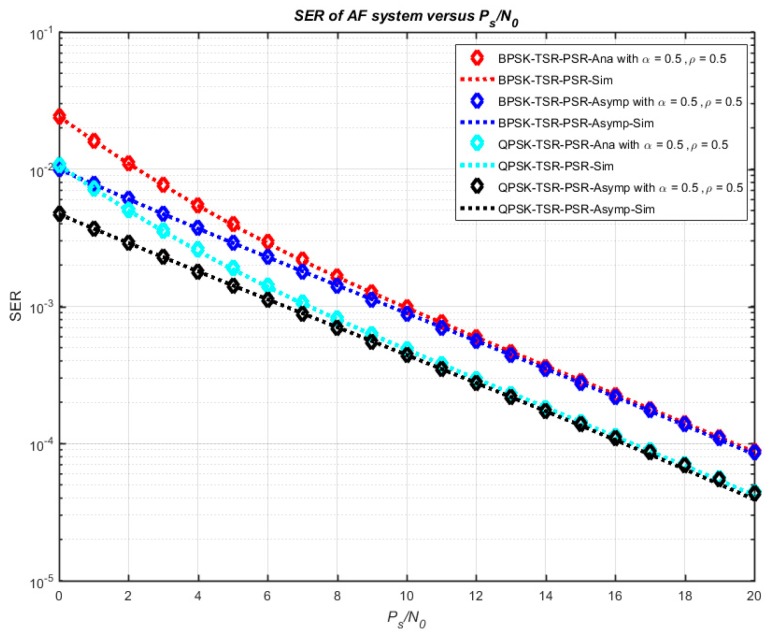
The exact and asymptotic SER versus *P_s_*/*N*_0_.

Figure 7 illustrates the numerical and simulation results of the system outage probability concerning *α* and *ρ* with *P_s_*/*N*_0_ = 10 dB. It is clearly shown that the outage probability increases with increasing *α* and *ρ*, and the minimum outage probability can be obtained with *α* = 0 and *ρ* = 1. Moreover, SER versus the ratio *P_s_*/*N*_0_ in three cases—TSR, PSR, and TSR–PSR—is shown in Figure 8. Furthermore, Figure 9 plots the comparison of the exact and asymptotic outage probability of three cases—TSR, PSR, and TSR–PSR—versus *P_s_*/*N*_0_. The results indicate that all the simulation and analytical values agreed well with each other.

## 5. Conclusions

In this research, the hybrid TSR–PSR alternate EH relay network over AF-based Rician fading channels is presented and investigated. The system model consists of one source, one destination and two alternative relays for signal transmission from the source to the destination. We derive the exact and asymptotic expressions of the outage probability and SER and investigate the influence of all system parameters on the system performance using the Monte Carlo simulation. The research results indicate that the alternative hybrid TSR–PSR has better performance in comparison with the TSR and PSR cases. The research results can provide essential recommendations for communication network research and practice directions.

## Figures and Tables

**Table 1:** All symbols used.

Symbol	Definition
0<η<1	Energy conversion efficiency
0≤α<1	Time-switching factor
0≤ρ<1	Power-splitting factor
*P_s_*/*N*_0_	Source-power-to-noise ratio
K	Rician K-factor
$\lambda_{1}$	Mean of ${\left|h_{1}\right|}^{2}$
$\lambda_{2}$	Mean of ${\left|g_{1}\right|}^{2}$
R	Source rate
*E_r_*_1_	Harvested energy at relay 1
*P_r_*_1_	Average transmit power of relay 1
*E_r_*_2_	Harvested energy at relay 2
*P_r_*_2_	Average transmit power of relay 2
*P_out_*	Outage probability
$\gamma_{e2e1}$	End to end signal to noise ratio
$K_{v}(•)$	Modified Bessel function of the second kind and *v*th order
Γ(•)	Gamma function
F(υ,β;γ;z)	Hypergeometric function
SER	Symbol error ratio
β	Amplifying factor
Q(t)	Gaussian Q-function
*P_s_*	Transmit power of the source
*T*	Total time of processing

**Table 2:** Simulation parameters.

Symbol	Values
0<η≤1	0.7
$\lambda_{1}$	1
$\lambda_{2}$	1
*P_s_*/*N*_0_	0:20 dB
*K*	3
R	0.5 bps

## Appendix A. Proof of Theorem 1—Exact Outage Probability

The outage probability of the model system can be calculated as

(A1) $$P_{out}^{1}=\mathrm{Pr}\left(\gamma_{e2e1}<\gamma_{th}\right)=\mathrm{Pr}\left[\frac{(1-\rho )P_{s}P_{r1}\varphi_{1}\varphi_{2}}{\varphi_{2}N_{0}+(1-\rho )P_{s}\varphi_{1}N_{0}}<\gamma_{th}\right]=\mathrm{Pr}\left[(1-\rho )P_{s}\varphi_{1}\left\{P_{r1}\varphi_{2}-\gamma_{th}N_{0}\right\}<\gamma_{th}\varphi_{2}N_{0}\right]$$

(A2) $$P_{out}^{1}=\mathrm{Pr}\left\{ \begin{array}{ll} \varphi_{1}<\frac{\gamma_{th}\varphi_{2}N_{0}}{P_{s}(1-\rho )\left[P_{r1}\varphi_{2}-\gamma_{th}N_{0}\right]} & ,\varphi_{2}>\frac{\gamma_{th}N_{0}}{P_{r1}}\\ 1 & ,\varphi_{2}\leq \frac{\gamma_{th}N_{0}}{P_{r1}} \end{array}\right.$$

where $\gamma_{th}=2^{2R}-1$ is threshold and R is the source rate.

The Equation (A2) can be rewritten as

(A3) $$P_{out}^{1}=\int_{0}^{\frac{\gamma_{th}N_{0}}{P_{r1}}}f_{\varphi_{2}}(\varphi_{2})d\varphi_{2}+\int_{\frac{\gamma_{th}N_{0}}{P_{r1}}}^{\infty }F_{\varphi_{1}}\left\{\frac{\gamma_{th}\varphi_{2}N_{0}}{P_{s}(1-\rho )\left[P_{r1}\varphi_{2}-\gamma_{th}N_{0}\right]}|\varphi_{2}\right\}f_{\varphi_{2}}(\varphi_{2})d\varphi_{2}$$

From (18), the outage probability can be formulated as the following

(A4) $$\begin{array}{l} P_{out}^{1}=\int_{0}^{\frac{\gamma_{th}N_{0}}{P_{r1}}}f_{\varphi_{2}}(\varphi_{2})d\varphi_{2}+\\ \int_{\frac{\gamma_{th}N_{0}}{P_{r1}}}^{\infty }\left\{1-\sum_{l=0}^{\infty }\sum_{n=0}^{l}\frac{K^{l}b^{n}e^{-K}}{l!n!}{\left(\frac{\gamma_{th}\varphi_{2}N_{0}}{P_{s}(1-\rho )\left[P_{r1}\varphi_{2}-\gamma_{th}N_{0}\right]}\right)}^{n}e^{-b\left(\frac{\gamma_{th}\varphi_{2}N_{0}}{P_{s}(1-\rho )\left[P_{r1}\varphi_{2}-\gamma_{th}N_{0}\right]}\right)}\right\}f_{\varphi_{2}}(\varphi_{2})d\varphi_{2} \end{array}$$

(A5) $$P_{out}^{1}=1-\int_{\frac{\gamma_{th}N_{0}}{P_{r1}}}^{\infty }\left\{\sum_{l=0}^{\infty }\sum_{n=0}^{l}\frac{K^{l}b^{n}e^{-K}}{l!n!}{\left(\frac{\gamma_{th}\varphi_{2}N_{0}}{P_{s}(1-\rho )\left[P_{r1}\varphi_{2}-\gamma_{th}N_{0}\right]}\right)}^{n}e^{-b\left(\frac{\gamma_{th}\varphi_{2}N_{0}}{P_{s}(1-\rho )\left[P_{r1}\varphi_{2}-\gamma_{th}N_{0}\right]}\right)}\right\}f_{\varphi_{2}}(\varphi_{2})d\varphi_{2}$$

Furthermore, from (17) we obtain

(A6) $$P_{out}^{1}=1-\int_{\frac{\gamma_{th}N_{0}}{P_{r1}}}^{\infty }\left\{\sum_{l=0}^{\infty }\sum_{n=0}^{l}\frac{K^{l}b^{n}e^{-K}}{l!n!}{\left(\frac{\gamma_{th}\varphi_{2}N_{0}}{P_{s}(1-\rho )\left[P_{r1}\varphi_{2}-\gamma_{th}N_{0}\right]}\right)}^{n}e^{-b\left(\frac{\gamma_{th}\varphi_{2}N_{0}}{P_{s}(1-\rho )\left[P_{r1}\varphi_{2}-\gamma_{th}N_{0}\right]}\right)}\right\}a\sum_{l=0}^{\infty }\frac{{\left(bK\right)}^{l}}{{\left(l!\right)}^{2}}x^{l}e^{-bx}d\varphi_{2}$$

(A7) $$P_{out}^{1}=1-\int_{\frac{\gamma_{th}N_{0}}{P_{r1}}}^{\infty }ae^{-K}\sum_{l=0}^{\infty }\sum_{m=0}^{\infty }\sum_{n=0}^{l}\frac{K^{l+m}b^{n+m}}{l!{\left(m!\right)}^{2}n!}{\left(\frac{\gamma_{th}\varphi_{2}N_{0}}{P_{s}(1-\rho )\left[P_{r1}\varphi_{2}-\gamma_{th}N_{0}\right]}\right)}^{n}e^{-b\left(\frac{\gamma_{th}\varphi_{2}N_{0}}{P_{s}(1-\rho )\left[P_{r1}\varphi_{2}-\gamma_{th}N_{0}\right]}\right)}\varphi_{2}^{m}e^{-b\varphi_{2}}d\varphi_{2}$$

By changing a variable $t=P_{r1}\varphi_{2}-\gamma_{th}N_{0}$ in to (25), we obtain

(A8) $$P_{out}^{1}=1-\frac{ae^{-K}}{P_{r1}}\int_{0}^{\infty }\sum_{l=0}^{\infty }\sum_{m=0}^{\infty }\sum_{n=0}^{l}\frac{K^{l+m}b^{n+m}}{l!{\left(m!\right)}^{2}n!}{\left(\frac{\gamma_{th}\left[\frac{t+\gamma_{th}N_{0}}{P_{r1}}\right]N_{0}}{P_{s}(1-\rho )t}\right)}^{n}e^{-b\left(\frac{\gamma_{th}\left[\frac{t+\gamma_{th}N_{0}}{P_{r1}}\right]N_{0}}{P_{s}(1-\rho )t}\right)}{\left[\frac{t+\gamma_{th}N_{0}}{P_{r1}}\right]}^{m}e^{-b\left[\frac{t+\gamma_{th}N_{0}}{P_{r1}}\right]}dt$$

(A9) $$P_{out}^{1}=1-\frac{ae^{-K}e^{-\frac{b\gamma_{th}N_{0}}{P_{r1}P_{s}(1-\rho )}}e^{-\frac{b\gamma_{th}N_{0}}{P_{r1}}}}{P_{r1}}\int_{0}^{\infty }\sum_{l=0}^{\infty }\sum_{m=0}^{\infty }\sum_{n=0}^{l}\frac{K^{l+m}b^{n+m}}{l!{\left(m!\right)}^{2}n!P_{r1}{}^{n+m}}{\left[\frac{\gamma_{th}N_{0}}{P_{s}(1-\rho )}\right]}^{n}t^{-n}e^{-\frac{b\gamma_{th}^{2}N_{0}^{2}}{P_{r1}P_{s}(1-\rho )t}}{\left[t+\gamma_{th}N_{0}\right]}^{n+m}e^{-\frac{bt}{P_{r1}}}dt$$

Now, by applying the equation ${\left(x+y\right)}^{n}=\sum_{k=0}^{n}\left(\begin{array}{c} n\\ k \end{array}\right)x^{n-k}y^{k}$ to (20), the outage probability can be demonstrated as follows:

(A10) $$\begin{array}{l} P_{out}^{1}=1-\frac{ae^{-K}e^{-\frac{b\gamma_{th}N_{0}}{P_{r1}P_{s}(1-\rho )}}e^{-\frac{b\gamma_{th}N_{0}}{P_{r1}}}}{P_{r1}}\sum_{l=0}^{\infty }\sum_{m=0}^{\infty }\sum_{n=0}^{l}\sum_{k=0}^{n+m}\frac{K^{l+m}b^{n+m}(n+m)!{\left(\gamma_{th}N_{0}\right)}^{n+k}}{l!{\left(m!\right)}^{2}n!k!(n+m-k)!P_{r1}{}^{n+m}}{\left[\frac{1}{P_{s}(1-\rho )}\right]}^{n}\\ \times \int_{0}^{\infty }t^{m-k}e^{-\frac{b\gamma_{th}^{2}N_{0}^{2}}{P_{r1}P_{s}(1-\rho )t}}e^{-\frac{bt}{P_{r1}}}dt \end{array}$$

Apply (3.471,9) of [32]:

(A11) $$\begin{array}{l} P_{out}^{1}=1-\frac{2ae^{-K}e^{-\frac{b\gamma_{th}N_{0}}{P_{r1}P_{s}(1-\rho )}}e^{-\frac{b\gamma_{th}N_{0}}{P_{r1}}}}{P_{r1}}\sum_{l=0}^{\infty }\sum_{m=0}^{\infty }\sum_{n=0}^{l}\sum_{k=0}^{n+m}\frac{K^{l+m}b^{n+m}{\left(\gamma_{th}N_{0}\right)}^{n+k}(n+m)!}{l!{\left(m!\right)}^{2}n!k!(n+m-k)!P_{r1}{}^{n+m}{\left[P_{s}(1-\rho )\right]}^{n}}\\ \times {\left(\frac{\gamma_{th}^{2}N_{0}^{2}}{P_{s}(1-\rho )}\right)}^{\frac{m-k+1}{2}}\times K_{m-k+1}\left(\frac{2b\gamma_{th}N_{0}}{P_{r1}\sqrt{P_{s}(1-\rho )}}\right) \end{array}$$

## Appendix B. Proof of Theorem 2—Exact SER Analysis

Substituting (17) into (26):

(A12) $$SER_{1}=\frac{\phi \sqrt{\theta }}{2\sqrt{\pi }}\int_{0}^{\infty }\frac{e^{-\theta x}}{\sqrt{x}}\left\{\begin{array}{l} 1-2ae^{-K}\sum_{l=0}^{\infty }\sum_{m=0}^{\infty }\sum_{n=0}^{l}\sum_{k=0}^{n+m}\frac{K^{l+m}b^{n+m}(n+m)!}{l!{\left(m!\right)}^{2}n!k!(n+m-k)!P_{r1}{}^{n+m+1}{\left[P_{s}(1-\rho )\right]}^{\frac{2n+m-k+1}{2}}}\\ \times {\left(xN_{0}\right)}^{n+m+1}\times e^{-\frac{bxN_{0}}{P_{r1}P_{s}(1-\rho )}}\times e^{-\frac{bxN_{0}}{P_{r1}}}\times K_{m-k+1}\left(\frac{2bxN_{0}}{P_{r1}\sqrt{P_{s}(1-\rho )}}\right) \end{array}\right\}dx$$

From (A12), we obtain:

(A13) $$I_{1}=\frac{\phi \sqrt{\theta }}{2\sqrt{\pi }}\int_{0}^{\infty }\frac{e^{-\theta x}}{\sqrt{x}}dx$$

and

(A14) $$\begin{array}{l} I_{2}=\frac{ae^{-K}\phi \sqrt{\theta }}{\sqrt{\pi }}\sum_{l=0}^{\infty }\sum_{m=0}^{\infty }\sum_{n=0}^{l}\sum_{k=0}^{n+m}\frac{K^{l+m}b^{n+m}(n+m)!{\left(N_{0}\right)}^{n+m+1}}{l!{\left(m!\right)}^{2}n!k!(n+m-k)!P_{r1}{}^{n+m+1}{\left[P_{s}(1-\rho )\right]}^{\frac{2n+m-k+1}{2}}}\\ \times \int_{0}^{\infty }{\left(x\right)}^{n+m+1}\times \frac{e^{-\theta x}}{\sqrt{x}}\times e^{-\frac{bxN_{0}}{P_{r1}P_{s}(1-\rho )}}\times e^{-\frac{bxN_{0}}{P_{r1}}}\times K_{m-k+1}\left(\frac{2bxN_{0}}{P_{r1}\sqrt{P_{s}(1-\rho )}}\right)dx \end{array}$$

Firstly, we consider $I_{1}=\frac{\phi \sqrt{\theta }}{2\sqrt{\pi }}\int_{0}^{\infty }\frac{e^{-\theta x}}{\sqrt{x}}dx$.

We use the table of integral Equation (3.361,1) in [32]:

(A15) $$I_{1}=\frac{\phi \sqrt{\theta }}{2\sqrt{\pi }}\times \frac{\sqrt{\pi }}{\sqrt{\theta }}=\frac{\phi }{2}$$

We consider

(A16) $$\begin{array}{l} I_{2}=\frac{ae^{-K}\phi \sqrt{\theta }}{\sqrt{\pi }}\sum_{l=0}^{\infty }\sum_{m=0}^{\infty }\sum_{n=0}^{l}\sum_{k=0}^{n+m}\frac{K^{l+m}b^{n+m}(n+m)!{\left(N_{0}\right)}^{n+m+1}}{l!{\left(m!\right)}^{2}n!k!(n+m-k)!P_{r1}{}^{n+m+1}{\left[P_{s}(1-\rho )\right]}^{\frac{2n+m-k+1}{2}}}\\ \times \int_{0}^{\infty }x^{n+m+1/2}\times e^{-x\left[\theta +\frac{bN_{0}}{P_{r1}P_{s}(1-\rho )}+\frac{bN_{0}}{P_{r1}}\right]}\times K_{m-k+1}\left(\frac{2bxN_{0}}{P_{r1}\sqrt{P_{s}(1-\rho )}}\right)dx \end{array}$$

(A17) $$J_{1}=\int_{0}^{\infty }x^{n+m+1/2}\times e^{-x\left[\theta +\frac{bN_{0}}{P_{r1}P_{s}(1-\rho )}+\frac{bN_{0}}{P_{r1}}\right]}\times K_{m-k+1}\left(\frac{2bxN_{0}}{P_{r1}\sqrt{P_{s}(1-\rho )}}\right)dx$$

We use the table of integral Equation (6.621,3) in [32]:

(A18) $$\begin{array}{l} J_{1}=\frac{\sqrt{\pi }{\left[\frac{4bN_{0}}{P_{r1}\sqrt{P_{s}(1-\rho )}}\right]}^{m-k+1}}{{\left[\theta +\frac{3bN_{0}}{P_{r1}\sqrt{P_{s}(1-\rho )}}+\frac{bN_{0}}{P_{r1}}\right]}^{n+2m-k+5/2}}\times \frac{\Gamma \left(n+2m-k+\frac{5}{2}\right)\Gamma \left(n+k+\frac{1}{2}\right)}{\Gamma \left(n+m+2\right)}\\ \times F\left(n+2m-k+\frac{5}{2},m-k+\frac{3}{2};n+m+2;\frac{\theta -\frac{bN_{0}}{P_{r1}\sqrt{P_{s}(1-\rho )}}+\frac{bN_{0}}{P_{r1}}}{\theta +\frac{3bN_{0}}{P_{r1}\sqrt{P_{s}(1-\rho )}}+\frac{bN_{0}}{P_{r1}}}\right) \end{array}$$

where Γ(•) is the gamma function, and F(υ,β;γ;z) is a hypergeometric function.

Then:

(A19) $$\begin{array}{l} I_{2}=\frac{ae^{-K}\phi \sqrt{\theta }}{\sqrt{\pi }}\sum_{l=0}^{\infty }\sum_{m=0}^{\infty }\sum_{n=0}^{l}\sum_{k=0}^{n+m}\frac{K^{l+m}b^{n+m}(n+m)!{\left(N_{0}\right)}^{n+m+1}}{l!{\left(m!\right)}^{2}n!k!(n+m-k)!P_{r1}{}^{n+m+1}{\left[P_{s}(1-\rho )\right]}^{\frac{2n+m-k+1}{2}}}\\ \times \frac{\sqrt{\pi }{\left[\frac{4bN_{0}}{P_{r1}\sqrt{P_{s}(1-\rho )}}\right]}^{m-k+1}}{{\left[\theta +\frac{3bN_{0}}{P_{r1}\sqrt{P_{s}(1-\rho )}}+\frac{bN_{0}}{P_{r1}}\right]}^{n+2m-k+5/2}}\times \frac{\Gamma \left(n+2m-k+\frac{5}{2}\right)\Gamma \left(n+k+\frac{1}{2}\right)}{\Gamma \left(n+m+2\right)}\\ \times F\left(n+2m-k+\frac{5}{2},m-k+\frac{3}{2};n+m+2;\frac{\theta -\frac{bN_{0}}{P_{r1}\sqrt{P_{s}(1-\rho )}}+\frac{bN_{0}}{P_{r1}}}{\theta +\frac{3bN_{0}}{P_{r1}\sqrt{P_{s}(1-\rho )}}+\frac{bN_{0}}{P_{r1}}}\right) \end{array}$$

(A20) $$\begin{array}{l} I_{2}=ae^{-K}\phi \sqrt{\theta }\sum_{l=0}^{\infty }\sum_{m=0}^{\infty }\sum_{n=0}^{l}\sum_{k=0}^{n+m}\frac{4^{m-k+1}K^{l+m}b^{n+2m-k+1}(n+m)!{\left(N_{0}\right)}^{n+2m-k+2}}{l!{\left(m!\right)}^{2}n!k!(n+m-k)!P_{r1}{}^{n+2m-k+2}{\left[P_{s}(1-\rho )\right]}^{\frac{2n+3m-3k+3}{2}}}\\ \times \frac{1}{\theta {\left[\phi +\frac{3bN_{0}}{P_{r1}\sqrt{P_{s}(1-\rho )}}+\frac{bN_{0}}{P_{r1}}\right]}^{n+2m-k+5/2}}\times \frac{\Gamma \left(n+2m-k+\frac{5}{2}\right)\Gamma \left(n+k+\frac{1}{2}\right)}{\Gamma \left(n+m+2\right)}\\ \times F\left(n+2m-k+\frac{5}{2},m-k+\frac{3}{2};n+m+2;\frac{\theta -\frac{bN_{0}}{P_{r1}\sqrt{P_{s}(1-\rho )}}+\frac{bN_{0}}{P_{r1}}}{\theta +\frac{3bN_{0}}{P_{r1}\sqrt{P_{s}(1-\rho )}}+\frac{bN_{0}}{P_{r1}}}\right) \end{array}$$

## Appendix C. Proof of Theorem 3—Asymptotic SER Analysis

We obtain

(A21) $$\begin{array}{cc} SER_{1}^{\infty } & =\frac{\phi \sqrt{\theta }}{2\sqrt{\pi }}\int_{0}^{\infty }\frac{e^{-\theta x}}{\sqrt{x}}\left\{1-\sum_{l=0}^{\infty }\sum_{n=0}^{l}\frac{K^{l}b^{n}e^{-K}}{l!n!}{\left(\frac{xN_{0}}{P_{r1}}\right)}^{n}e^{-\frac{bxN_{0}}{P_{r1}}}\right\}dx\\ & =\frac{\phi \sqrt{\theta }}{2\sqrt{\pi }}\int_{0}^{\infty }\frac{e^{-\theta x}}{\sqrt{x}}dx-\frac{e^{-K}\phi \sqrt{\theta }}{2\sqrt{\pi }}\sum_{l=0}^{\infty }\sum_{n=0}^{l}\frac{K^{l}b^{n}}{l!n!}{\left(\frac{N_{0}}{P_{r1}}\right)}^{n}\int_{0}^{\infty }x^{n}\times \frac{e^{-\theta x}}{\sqrt{x}}\times e^{-\frac{bxN_{0}}{P_{r1}}}dx \end{array}$$

We consider

(A22) $$I_{3}=\int_{0}^{\infty }x^{n}\times \frac{e^{-\theta x}}{\sqrt{x}}\times e^{-\frac{bxN_{0}}{P_{r1}}}dx=\int_{0}^{\infty }x^{n-1/2}\times e^{-x\left(\theta +\frac{bN_{0}}{P_{r1}}\right)}dx$$

We use the table of integral Equation (3.381,4) in [32]:

(A23) $$I_{3}={\left(\theta +\frac{bN_{0}}{P_{r1}}\right)}^{-n-1/2}\Gamma \left(n+\frac{1}{2}\right)$$

where Γ(•) is the gamma function.
